# Neuronal representation of bimanual arm motor imagery in the motor cortex of a tetraplegia human, a pilot study

**DOI:** 10.3389/fnins.2023.1133928

**Published:** 2023-03-01

**Authors:** Dongrong Lai, Zijun Wan, Jiafan Lin, Li Pan, Feixiao Ren, Junming Zhu, Jianmin Zhang, Yueming Wang, Yaoyao Hao, Kedi Xu

**Affiliations:** ^1^Qiushi Academy for Advanced Studies, Zhejiang University, Hangzhou, China; ^2^Key Laboratory of Biomedical Engineering of Education Ministry, Department of Biomedical Engineering, Zhejiang University, Hangzhou, China; ^3^Zhejiang Provincial Key Laboratory of Cardio-Cerebral Vascular Detection Technology and Medicinal Effectiveness Appraisal, Zhejiang University, Hangzhou, China; ^4^College of Computer Science and Technology, Zhejiang University, Hangzhou, China; ^5^Department of Neurosurgery, The Second Affiliated Hospital, School of Medicine, Zhejiang University, Hangzhou, China; ^6^MOE Frontier Science Center for Brain Science and Brain-Machine Integration, Zhejiang University, Hangzhou, China

**Keywords:** motor imagery, brain-computer interface (BCI), primary motor cortex (M1), bimanual arm movement, neuronal representation

## Abstract

**Introduction:**

How the human brain coordinates bimanual movements is not well-established.

**Methods:**

Here, we recorded neural signals from a paralyzed individual’s left motor cortex during both unimanual and bimanual motor imagery tasks and quantified the representational interaction between arms by analyzing the tuning parameters of each neuron.

**Results:**

We found a similar proportion of neurons preferring each arm during unimanual movements, however, when switching to bimanual movements, the proportion of contralateral preference increased to 71.8%, indicating contralateral lateralization. We also observed a decorrelation process for each arm’s representation across the unimanual and bimanual tasks. We further confined that these changes in bilateral relationships are mainly caused by the alteration of tuning parameters, such as the increased bilateral preferred direction (PD) shifts and the significant suppression in bilateral modulation depths (MDs), especially the ipsilateral side.

**Discussion:**

These results contribute to the knowledge of bimanual coordination and thus the design of cutting-edge bimanual brain-computer interfaces.

## 1. Introduction

Brain-computer interfaces (BCIs) are sophisticated artificial systems that aim to restore upper arms functionality in persons with tetraplegia (Lebedev and Nicolelis, 2017). Under intracortical BCIs, the participants can control external devices, such as computer cursors (Hochberg et al., 2006; Simeral et al., 2011; Pandarinath et al., 2017) and robotic limbs (Hochberg et al., 2012; Collinger et al., 2013; Wodlinger et al., 2015; Flesher et al., 2021; Handelman et al., 2022), to perform some activities of daily living. The frontiers of BCIs demonstrated the use of multi-area neural signals to enable the participant to perform the volitional self-feeding task with bimanual arms (Handelman et al., 2022). To further advance BCI technology in clinical applications, several neurophysiological studies have focused on bimanual arm movements, which studies knowledge about bimanual coordination in the brain (Mooshagian and Snyder, 2018; Mooshagian et al., 2018; Cross et al., 2020).

It’s widely convinced that motor neurons in the primary motor cortex (M1) area encoded movement kinematics of the contralateral arm, such as velocity and target position (Georgopoulos et al., 1982; Truccolo et al., 2008; Bundy et al., 2018). Interestingly, the directional tuning to the ipsilateral arm was also observed in motor neurons during ipsilateral movements and most neurons were activated when using either the ipsilateral or contralateral arm (Tanji et al., 1988; Donchin et al., 1998; Kermadi et al., 1998), suggesting the mixing coding of bilateral movements in M1. One important question is whether neural representation varied between the contralateral and ipsilateral movements. Many neurophysiological studies with non-human primates reported that a large number of M1 neurons responded similarly using either arm or thus the outputs in M1 were not lateralized (Kermadi et al., 1998; Donchin et al., 2002; Ames and Churchland, 2019). Some of them observed strongly correlated tunings for the ipsilateral and contralateral movements in a group of M1 neurons (Steinberg et al., 2002; Rokni et al., 2003) which can be attributed to similar tuning parameters (Steinberg et al., 2002; Cisek et al., 2003; Rokni et al., 2003). However, others observed that only a small number of neurons exhibited a strong bilateral correlation (Ames and Churchland, 2019).

It has been shown that M1 neurons exhibited bimanual-related activity and had a complex directional tuning to the bimanual movements (Donchin et al., 1998, 2002; Kermadi et al., 1998; Cisek et al., 2003; Rokni et al., 2003). Studies on the neural representation of bimanual movements proposed that no significant lateralization was found in M1 during unimanual arm movement (Donchin et al., 2002; Ames and Churchland, 2019), yet the lateralized responses were observed in many M1 neurons when simultaneously using both arms (Donchin et al., 2002; Steinberg et al., 2002; Rokni et al., 2003; Ames and Churchland, 2019). Some of studies have suggested that such changes in lateralization were mainly attributed to the high similarity between bimanual and contralateral representations and the modified ipsilateral tuning (Donchin et al., 2002; Steinberg et al., 2002; Rokni et al., 2003), like the largely suppressed modulation depths (MDs) and/or preferred direction (PD) shifts on the ipsilateral side. However, a more detailed analysis with a postural perturbation task (Cross et al., 2020) further explored the relationship between arms and argued that most neurons maintained the PDs for each arm across the unimanual and bimanual movements. They also showed that the MDs for both arms were suppressed to similar degrees during bimanual movements. By investigating the directional tuning of bimanual movements, researchers can describe how M1 units represent the bimanual movements and, consequently, fit the neural activity to decoding models that enable bimanual control in monkey (Ifft et al., 2013) and human (Handelman et al., 2022).

It’s still debated how neural activities interact for bimanual movements in M1. Moreover, most of these studies were conducted with non-human primates. We then asked whether the hypotheses could also apply to those in humans. In this study, we set up a human-based BCI system and recorded neural activities in the participant’s left M1 area during unimanual and bimanual motor imagery tasks. We supported that the M1 outputs are not lateralized during unimanual movements, yet changed to prefer the contralateral arm during bimanual movements. Then, we analyzed the neural activities of bilateral arms and showed a decorrelation process between ipsilateral and contralateral arms when switching to bimanual movements. Furthermore, we suggested that such changes are mainly due to the modification of tuning parameters.

## 2. Materials and methods

### 2.1. Participant

The participant was a 72 year-old right-handed man with a C4-level spinal cord injury, leading to his sensory and motor disability below the shoulder. Surgical procedures, postoperative nursing care, and data acquisition were performed 2 years after the injury at the Second Affiliated Hospital Zhejiang University School of Medicine (Hangzhou, Zhejiang, China). Data analysis was conducted at Zhejiang University (Hangzhou, Zhejiang, China).

### 2.2. Experimental setup and data recording

**FIGURE 1:**
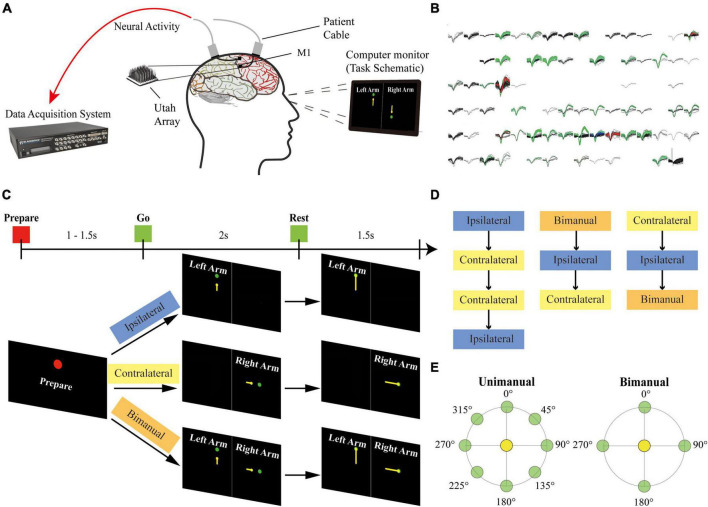
Setup of the experiment. **(A)** The participant was implanted with two 96-channel microelectrode arrays in his left M1 area. The neural signal was recorded with the NeuroPort system from the arrays during the motor imagery task. **(B)** Example panel of the spike activities for 96-channel arrays during a recording session. Each sub-panel displayed the spikes for a channel, and each color except for black indicated a sorted neuron. **(C)** Task schematic. The participant performed motor imagery for the center-out reaching task with the ipsilateral arm, the contralateral arm, and the bimanual arms in different blocks. Each trial contains a Prepare phase, a Go phase for imagery of arm movements from the center target (yellow circles) to the peripheral targets (green circles), and a Rest phase. Text and colored shapes were displayed as the movement cues. **(D)** Blocks of different movement types recorded on each day were presented in a pseudorandom order. **(E)** Overview of 8 peripheral targets on a circle for the unimanual trials and 4 peripheral targets for each arm for the bimanual trials.

Two 96-channel Utah microelectrode arrays (Blackrock Microsystems, Salt Lake City, UT, USA) were implanted into his arm and hand knob M1 areas on 27 August 2019. During the recording sessions, two patient cables ran from the connectors on his head to the NeuroPort data acquisition system (Blackrock Microsystems). The motor imagery tasks were displayed on the computer monitor in front of the participant (Figure 1A).

Single-unit activity has been recorded from electrodes for this study since day 258 after surgery. Sessions of ipsilateral, contralateral, and bimanual arm movements were performed across 3 days. Two more sessions only for the ipsilateral and contralateral arm movements were collected on other 2 days.

### 2.3. Paradigm

As shown in Figure 1C, at the beginning of a trial, the participant received the acoustic and visual cues to prepare for 1,000–1,500 ms (Prepare phase). In the next 2,000 ms, a yellow circle appeared at the center position as the start position and then moved to the peripheral targets. The participant was cued to imagine moving the arm along the yellow trajectory (Go phase), and then to relax for 1,500 ms when reached the peripheral targets (Rest phase).

A single session consisted of ipsilateral (left), contralateral (right), and bimanual blocks. Blocks were presented in pseudorandom order. Within a block, 8 repetitions of one set of movements were performed. Each set of movements contains 8 possible target positions for ipsilateral and contralateral blocks or 16 possible configurations for bimanual blocks (Figure 1E).

### 2.4. Data analysis

#### 2.4.1. Single-unit analysis

Single units were sorted for each session (Figure 1B). Spike counts were recorded, binned with 20 ms bins, converted into the firing rate (Hz), and then smoothed with a 300 ms Gaussian kernel (Ames and Churchland, 2019; Rastogi et al., 2020; Willett et al., 2020). Neurons with mean firing rates over 1 Hz over all blocks performed on a day were preselected to have a reasonable estimate of the directional modulation (Willett et al., 2020).

The single-neuron activity was first analyzed by peri-event time histogram (PETH). Single-unit PETH of neuronal discharge activity, aligned with the target onset in the Go phase, was constructed by mean firing rates for each trial type, respectively (Georgopoulos et al., 1982; Donchin et al., 2002). We then computed the baseline firing rate for each neuron, defined as the mean firing rate during the Prepare phase. Each neuron’s neural response for a specific target configuration was measured as the difference between the mean firing rate during the Go phase and the baseline (Donchin et al., 2002; Rokni et al., 2003). After the neural response was calculated, the modulation ratios were determined by the ratio between the absolute neural response and the standard deviations (SDs) of the baseline. We considered a unit to be task-related for the arm used if the modulation ratio was over 1 for at least a target configuration of the movement (Tanji et al., 1988; Ma et al., 2017).

#### 2.4.2. Arm preference analysis

To assess the lateral preference for each neuron, we compared the maximum absolute neural response for the contralateral arm to that for the ipsilateral arm. The strength of lateralization for a neuron was defined as the contralateral preference index (Donchin et al., 2002; Ames and Churchland, 2019; Gardner et al., 2022):


(1) $$C\,o\,n\,t\,r\,a\,l\,a\,t\,e\,r\,a\,l\,P\,r\,e\,f\,e\,r\,e\,n\,c\,e\,I\,n\,d\,e\,x=\frac{M\,a\,x\,\left(\left|R\,e\,s\,p\,o\,n\,s\,e\right|\;,\;C\,o\,n\,t\,r\,a\right)-M\,a\,x\,\left(\left|R\,e\,s\,p\,o\,n\,s\,e\right|\;,\;I\,p\,s\,i\right)}{M\,a\,x\,\left(\left|R\,e\,s\,p\,o\,n\,s\,e\right|\;,\;C\,o\,n\,t\,r\,a\right)+M\,a\,x\,\left(\left|R\,e\,s\,p\,o\,n\,s\,e\right|\;,\;I\,p\,s\,i\right)}$$

Here, a unit only modulates with the contralateral arm movements if the index is 1 while it only modulates with the ipsilateral arm movements if the index is −1. The neural response of a unit is identical regardless of the arm used if the index is 0.

We then assessed whether the outputs of M1 ensembles were lateralized during the unimanual and bimanual movements. A paired *t*-test was used to compare the distribution of preference index across neurons to the null distribution. It’s also used to quantify whether a statistically significant change existed in the lateralization for M1 ensembles across the unimanual versus bimanual movements. The statistical significance was defined as *p-*values < 0.05.

#### 2.4.3. Bilateral correlation analysis

We characterized each neuron’s relationship between the contralateral and ipsilateral arm during the unimanual and bimanual tasks. A neuron’s relationship between arms was defined as the Pearson’s correlation coefficient between the mean firing rates of each target configuration (i.e., tuning curve) for the contralateral and ipsilateral arm movements. As shown in Figure 1E, two groups of movement directions were analyzed: for the unimanual trials, each arm movement in 8 directions (Unimanual-8-direction) and 4 directions (Unimanual-4-direction) corresponding to those of the bimanual trials (Bimanual-4-direction). For M1 ensembles, we used the *t*-test to compare the means of distributions of correlation coefficients (*cc*) for both unimanual and bimanual tasks to the null distribution. *p*-values < 0.05 was used to define statistical significance.

#### 2.4.4. Tuning parameters

We then further quantified the changes in correlation by analyzing the directional tuning of ipsilateral and contralateral movements across unimanual versus bimanual tasks. The neuron’s characteristics of directional tuning were quantified as tuning parameters, including both the MDs and the PDs (Georgopoulos et al., 1982; Donchin et al., 2002; Rokni et al., 2003; Ifft et al., 2013; Cross et al., 2020). The neuron’s MD of each arm movement was defined as the difference between the maximum and minimum neural responses. We used the *t*-test to compare the means of distributions of MDs across unimanual versus bimanual tasks. The neuron’s PD of each arm movement was determined by the movement direction with the maximum neural response. We computed the difference between contralateral and ipsilateral PDs (ΔPD) across unimanual versus bimanual tasks. Both 8-direction and 4-direction data were analyzed for the unimanual task.

#### 2.4.5. Neural decoding using support vector machines

To extract the neural pattern from the population-level responses, we used the linear Support Vector Machine classifiers (SVMs) on the neural responses to classify each trial’s movement direction for each session. Two SVM classifiers were trained for a unimanual session to classify the movement directions for each arm; one SVM classifier was used to decode the 16 configurations of targets for a bimanual session. To further evaluate whether the outputs of M1 ensembles were lateralized in the bimanual task, for each bimanual session, we trained two more SVM classifiers for the 4 movement directions of bimanual contralateral and ipsilateral arms, respectively. We used leave-one-out cross-validation, with each iteration using a different trial for testing and the rest for training. The prediction accuracy of each movement type was computed with the Top-N approach. The paired *t*-test was used to judge whether there were statistically significant differences between the prediction accuracy for the two arm movements. We used *p*-values < 0.05 to define statistical significance.

## 3. Results

As shown in Figure 1, we recorded neural signals from the array implanted in the arm area of left M1 while the patient performed both unimanual and bimanual motor imagery tasks. The other array implanted in the hand knob area was not included due to a lack of single units. The patient randomly performed ipsilateral (left), contralateral (right), and bimanual tasks for 3 sessions. Two extra sessions only with ipsilateral and contralateral arm movements were recorded for offline classification.

**FIGURE 2:**
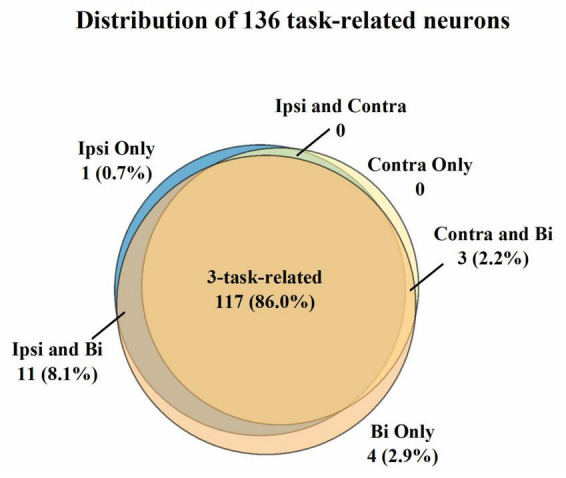
Classification of the 136 neurons with the mean firing rate > 1 Hz across three tasks. A neuron was judged to be task-related to a specific movement if its modulation ratio was higher than one during this specific movement. Most neurons (86.0%) were considered 3-task-related. Further analysis was compiled from the neural activity of 3-task-related neurons.

**FIGURE 3:**
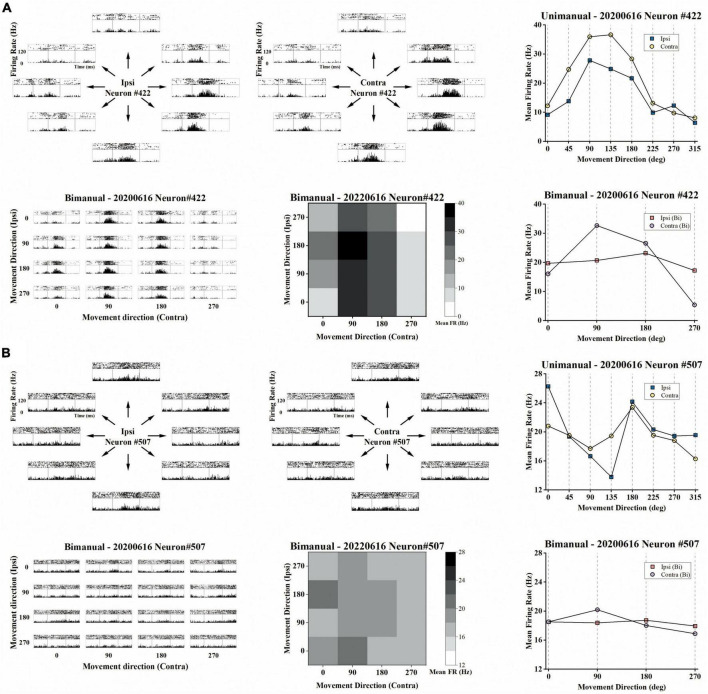
Example of the representative neurons in the unimanual and bimanual tasks. **(A)** A typical neuron with a contralateral preference in both the unimanual and bimanual tasks. It also showed strongly correlated activity for each arm movement which can be quantified as the high correlation coefficient (*cc* = 0.95) between their tuning curves. When switching to bimanual movements, it remained strongly correlated between arms (*cc* = 0.89) even though substantive changes were observed in both MDs and PDs of ipsilateral-related activity. Top row the peri-event time histograms (PETHs) during ipsilateral (left column) and contralateral movements (middle column) performed on the same day. Each subplot of PETHs was aligned on the appearance of the go cue (left gray line in each subplot). The averaged firing rate in the unimanual task was depicted in the right column, for 8 movement directions of ipsilateral trials (blue squares) and of contralateral trials (yellow dots); Bottom row same as in the top row, PETHs for the same neuron in the bimanual task (left column). The average firing rate in the bimanual task was depicted in the heatmap in the middle column and the line chart in the right column. **(B)** The other type of neuron with ipsilateral preference during unimanual movements but it inverted preference during bimanual movements. The representation of ipsilateral and contralateral arm movements was decorrelated with decreased *cc* that changed from 0.57 to 0.22. These changes can be attributed to the PD shifts and great suppression in bilateral sides.

For the three sessions’ data, we first selected 136 neurons with mean firing rates over 1 Hz across the unimanual and bimanual sessions performed each day (Figure 2). Most of them (117/136, 86%) like neurons in Figure 3 showed a significant increase in discharge frequency within the Go phase for at least a specific movement direction for each task and were classified as 3-task-related neurons. We then investigated how these neurons changed their firing pattern across these three tasks and how a single brain region represents bimanual arm movements.

### 3.1. Neuronal responses biased to the contralateral arm during bimanual movements

**FIGURE 4:**
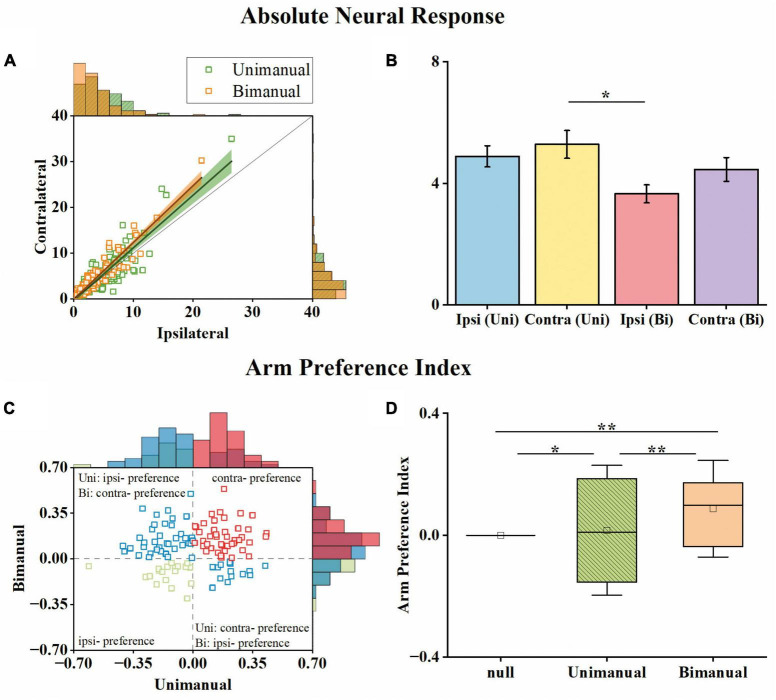
M1 units had similar neural responses for the ipsilateral and contralateral arm movements in the unimanual task but changed to prefer the contralateral arm in the bimanual task. **(A)** Scatter plot of the maximum absolute responses of the neurons during the ipsilateral versus contralateral arm movements. The blue squares plot the 117 3-task-related neurons in the unimanual task. The lines with the shaded regions represent the best fit line and 95% confidence interval for unimanual (*y* = 1.15x–0.34, *R*^2^ = 0.77; green) and bimanual (*y* = 1.24x–0.08, *R^2^* = 0.88; orange) movements. **(B)** Means of the maximum absolute responses for the unimanual ipsilateral (blue), unimanual contralateral (yellow), bimanual ipsilateral (red), and bimanual contralateral movements (purple). Bars and error bars depict the means ± SD. **(C)** Scatter plot and histogram of the arm preference index of the neurons during the unimanual versus bimanual arm movements. Each square represents a single neuron. **(D)** Boxplots of the median, the 25th, and 75th percentiles of the contralateral arm preference index for the data in panel **(C)**. The squares represent the mean. Error bars indicate the SD. *p < 0.05, **p < 0.01, *t*-test.

To determine whether lateralized neuronal representation exists, we computed the arm preference index for each neuron (see Section “2. Materials and methods”) based on their changes in neural responses across contralateral and ipsilateral movements. As shown in Figure 4A, we compared each neuron’s maximum absolute neural responses during contralateral and ipsilateral movements for both unimanual and bimanual tasks. In general, the responses for ipsilateral and contralateral movements were linearly correlated for both conditions. However, the response is more biased to the contralateral side in the bimanual task than in the unimanual task (slope = 1.24 for the bimanual task versus slope = 1.15 for the unimanual task). The slightly stronger neural response when using the contralateral versus ipsilateral arm during both tasks was also confirmed at the population level as shown in Figure 4B, which depicts the mean neural responses across neurons for each arm during both tasks. We found the ipsilateral to contralateral ratio was 92.4% during the unimanual task but decreased to 82.1% during the bimanual task. We calculated the arm preference index for each neuron, which was defined as the difference between the maximum absolute neural responses during contralateral versus ipsilateral movements, and plotted them in Figure 4C. As the distribution shown in Figure 4C, among the 117 neurons, nearly an equal number of neurons are with ipsilateral versus contralateral preference (58 versus 59 neurons) during unimanual movements, while the number of neurons with a contralateral preference increased to 84 (71.8%) in the bimanual task. The averaged preference index (Figure 4D) for M1 ensembles was close to zero during unimanual movements while it was significantly different from zero during bimanual movements (Unimanual: *mean* = 0.01, *median* = 0.02, *p* > 0.01 for unimanual movements and null distribution; Bimanual: *mean* = 0.10, *median* = 0.09, *p* < 0.01 for bimanual movements and null distribution; *t*-test). These results revealed that the M1 outputs were not lateralized during unimanual movements yet changed to prefer the contralateral arm during bimanual movements.

We then investigated how single neurons altered their preference across unimanual and bimanual movements. We found a substantial of neurons (38.5%) with a contralateral preference during unimanual movements, which remained the preference when switching to bimanual movements (Figure 4C, upper right quadrant). An example neuron of this type is shown in Figure 3A, which illustrated the contralateral preference during both unimanual movement (top row) and bimanual movement (bottom row). Actually, the ipsilateral modulation was suppressed almost to zero during bimanual movement while the representation for the contralateral movement was largely unchanged. Another commonly observed neuron type was with an ipsilateral preference during unimanual movements but exhibited an inverted preference during bimanual movements (33.3%, Figure 4C, upper left quadrant). An example of this type is shown in Figure 3B, which showed ipsilateral preference during unimanual movements (top row) but changed to contralateral preference during bimanual movements (bottom row). As shown in its tuning curves, neural modulation was larger when using the ipsilateral arm than using the contralateral arm in the unimanual task. However, large suppression was found in bilateral arm modulations in the bimanual task, especially for the ipsilateral side which decreased almost to zero. We also found a small group of neurons with an ipsilateral preference for both tasks (16.2%, Figure 4C, lower left quadrant). Only a small portion of neurons preferred the contralateral arm during the unimanual movements but inverted the preference to ipsilateral when using both arms simultaneously (12.0%, Figure 4C, lower right quadrant). These results indicate that a large population of neurons with ipsilateral preference during unimanual movements switched to the contralateral side during bimanual movements, which could be the reason why the neural ensemble representation is biased to the contralateral side during bimanual movements.

### 3.2. Decoding analysis revealed the same contralateral preference during bimanual movements

**FIGURE 5:**
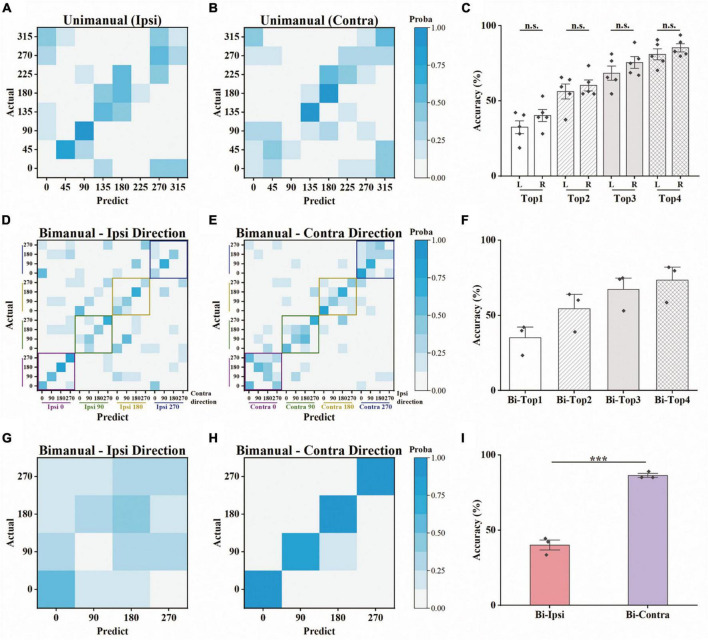
Offline discrete prediction for the unimanual and bimanual center-out tasks. **(A)** The support vector machine classifiers were used to classify each trial’s movement direction using the neural responses. Example confusion matrix of offline prediction results on an ipsilateral block. Each square (i, j) in the matrix is colored by the percentage of trials that target j was classified as target i. **(B)** The same as in panel **(A)**, showing results from a contralateral block performed on the same day. **(C)** Top-N accuracy of unimanual trials obtained by the SVM classifiers, for each ipsilateral and contralateral block, respectively. Each diamond represents the mean prediction performance for a block. **(D,E)** As in panels **(A,B)**, for the classification results predicted by another SVM classifier for a bimanual block. The confusion matrices were shown according to the direction of the ipsilateral and contralateral arm movements. **(F)** As in panel **(C)**, for the bimanual center-out trials. **(G)** Confusion matrix for the same bimanual block in panels **(D,E)**, using the SVM classifier with a 4D output for the direction of ipsilateral arm. **(H)** As in panel **(G)**, for the direction of contralateral arm. **(I)** As in panels **(C,F)**, for the mean prediction performance of the SVM classifiers with 4D output for the bimanual trials. The classifiers were trained on all the selected neurons for each block. ***p < 0.001, *t*-test.

We further asked whether the amount of information contained in the population was also biased toward the contralateral arm during bimanual movement. To that end, we employed a decoding analysis (see Section “2. Materials and methods”), in which the information contained was quantified as the prediction accuracy for a specific movement type. For each block of a movement type, we trained a linear SVM classifier to predict the target positions based on neural responses. For unimanual movement, the decoding results for example ipsilateral and contralateral blocks are shown in Figures 5A, B, respectively. Quantitatively, there is no significant difference between the prediction accuracies for ipsilateral and contralateral movement, regardless of whether top-1 to top-4 accuracies were considered (Figure 5C and Table 1). These results are consistent with the population proportional results shown above. We then used the same approach to assess the population-level information for the bimanual task. For bimanual movement, we plotted the prediction results on an example block according to the movement directions of the ipsilateral (Figure 5D) and contralateral arms (Figure 5E). Prediction accuracies across bimanual blocks were shown in Figure 5F. We noticed that the classification in each group of ipsilateral directions (the small squares in Figure 5D) was much better than the groups with the same contralateral directions (the small squares in Figure 5E), indicating the main contribution of the separation was from the contralateral arm. To further quantify the evidence, we trained two more classifiers for each bimanual block, one for predicting ipsilateral and one for contralateral directions. One example prediction result for the bimanual task showed much better prediction performance for the contralateral arm (comparing Figure 5G for ipsi-direction and Figure 5H for contra-direction). The prediction accuracy for the bimanual contralateral arm achieved 86.46% while the accuracy for the bimanual ipsilateral arm was only 40.10% (Figure 5I, *p* < 0.001, *t*-test). These results demonstrated that population-level signals contained information related to the movements of bilateral arms in the unimanual task, but mainly represent the information related to the movements of the contralateral arm when using both arms simultaneously, suggesting the contralateral preference of M1 population-level signals during bimanual movements.

**TABLE 1:** Offline prediction accuracy (*mean* ± SD) using support vector machines (SVMs) for the unimanual and bimanual tasks.

Top-N accuracy	Ipsilateral	Contralateral	16 configurations	Ipsi-direction	Contra-direction
Top-1	32.50 ± 9.59%	40.31 ± 8.93%	35.16 ± 10.22%	40.10 ± 5.76%	86.46 ± 2.26%
Top-2	56.25 ± 11.32%	60.29 ± 8.03%	54.43 ± 13.45%		
Top-3	68.44 ± 10.56%	75.63 ± 8.74%	67.45 ± 12.41%		
Top-4	80.94 ± 8.22%	85.31 ± 5.91%	73.44 ± 12.91%		

### 3.3. Strong correlations disappeared when switching to bimanual movements

**FIGURE 6:**
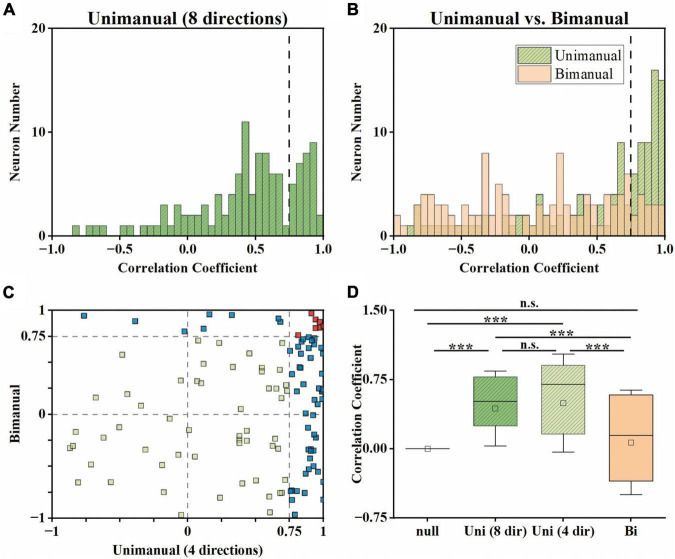
Neural activity for ipsilateral and contralateral arm movements was decorrelated across the unimanual and bimanual tasks. **(A)** For each neuron, we computed the *cc* between the tuning curves for 8 movement directions of each arm in the unimanual task. **(B)** Same as in panel **(A)**, but for the 4-direction data in the unimanual (green) and bimanual (orange) tasks. **(C)** Scatter plot of the *cc* in panel **(B)** for each neuron. 47.0% (55/117) of the neurons had a strong positive correlation (*cc* > 0.75) between the tuning curves for the two arms in the unimanual task while it declined to 12.8% (15/117) in the bimanual task. **(D)** Boxplots illustrated the median, the 25th, and 75th percentiles, and outliers outside the extremes (black diamonds) for the *cc* in panels **(A,B)**. The squares represent the mean. Error bars indicate the SD. Overall, these results revealed that the strong correlation between the ipsilateral- and contralateral-related activity disappeared in the unimanual versus bimanual tasks. ***p < 0.001, *t*-test.

Although the neural population was biased to contralateral encoding during bimanual movements, the ipsilateral movement was still represented, which can be indicated by the above chance level decoding accuracy (Figure 5I). We then wondered if single units changed their relationship between bilateral arm representations across unimanual and bimanual tasks, which was quantified as the correlation coefficient (*cc*) between their tuning curves for both arms. As shown by the example neurons in Figure 3, the tuning curves for ipsilateral and contralateral movement were usually well-correlated in unimanual tasks (*cc* = 0.95 and 0.57 for the top row in Figures 3A, B respectively). This is also true for the population as shown in Figures 6A, B, where the distribution of the *cc* skewed mostly rightward; around 47.0% of neurons in Figure 6B had a strong positive correlation (*cc* > 0.75) during the unimanual task, which indicated congruent directional tuning between two arms. However, when switched to the bimanual task, the correlation of bilateral tunings sharply decreased. For example, the *cc* of the neuron shown in the bottom row of Figure 3B reduced to 0.22. At the population level, the skewed distribution significantly changed to uniform as shown in the scatter plot in Figure 6C and boxplots in Figure 6D (Unimanual-8-direction, *mean* = 0.43, *median* = 0.51; Unimanual-4-direction, *mean* = 0.41, *median* = 0.70; Bimanual: *mean* = 0.07, *median* = 0.15, *t*-test, *p* < 0.001); only 12.8% of neurons with a strong positive correlation and no significant difference was found between the distribution for bimanual movements and the null distribution (*t*-test, *p* > 0.05). The above results demonstrated a decorrelation process between ipsilateral and contralateral arm movements for M1 ensembles when switching to bimanual movements.

### 3.4. Decorrelation between arms can be attributed to the alternation in tuning parameters

The above results showed that the tuning curves for each arm were decorrelated in the bimanual task. Intuitively, the decorrelation for neurons in Figure 3 are mostly attributed to the great suppression in ipsilateral modulation. Actually, shifts in directional tuning also existed on either the ipsilateral or the contralateral side like the neuron shown in Figure 3B. We further characterized the relationship between decorrelation and the changes in tuning parameters, including both MDs and PDs for each neuron.

**FIGURE 7:**
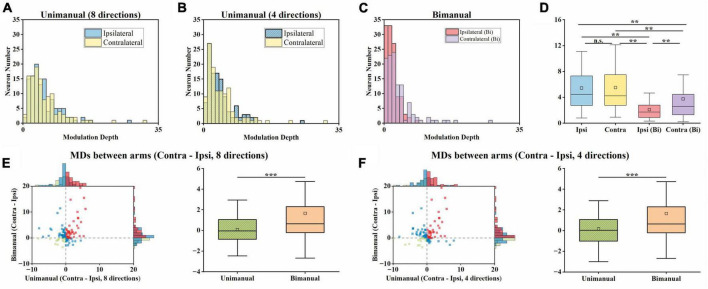
Both the ipsilateral and contralateral modulation depths (MDs) were significantly suppressed across unimanual and bimanual tasks, especially the ipsilateral side. **(A)** Distribution of MDs for ipsilateral and contralateral arms during unimanual movements, for unimanual-8-direction data. **(B)** Same as in panel **(A)**, but for the 4-direction data. **(C)** Same as in panels **(A,B)**, but for bimanual movements. **(D)** Boxplots of MDs across neurons. **(E)** Comparison of MDs between arms during the unimanual versus bimanual movements for unimanual-8-direction and bimanual data. **(F)** Similar to panel **(E)**, but for unimanual-4-direction and bimanual data. The significant suppression of the MDs of both arms was observed during the bimanual versus unimanual task. While the distributions of MDs for both arms were similar during the unimanual task, the shifts in MDs during the bimanual task led to the larger MDs in the contralateral versus ipsilateral sides in the bimanual task. **p < 0.01, ***p < 0.001, *t*-test.

For unimanual MDs, the example neurons in Figure 3A depicted a strong positive correlation between the tuning curves of both arms due to the similar bilateral MDs (*cc* = 0.95, ipsilateral MD = 27.03 versus contralateral MD = 31.32 during unimanual movements; the top row in Figure 3A). In contrast, other neurons like the one in Figure 3B differed significantly; the ipsilateral MD was almost 2 times greater than contralateral MD (ipsilateral MD = 15.92 versus contralateral MD = 8.01 in the unimanual movements; the top row in Figure 3B). In general, the distributions for the ipsilateral and contralateral MDs are highly overlapped in the unimanual task as shown in Figures 7A, B. When switched to the bimanual task, both distributions shifted leftward (i.e., lower MDs) relative to those for unimanual movements, especially for the ipsilateral side (Figure 7C). This was quantified in Figure 7D, in which the average MDs showed an overall significant decrease for bimanual movements (Unimanual-4-direction: *mean* = 4.40 and 4.58, *median* = 3.27 and 3.36, for ipsilateral and contralateral arms; Bimanual: *mean* = 2.10 and 3.76, *median* = 1.73 and 2.60, for ipsilateral and contralateral arms, *t*-test, *p* < 0.001).

We then examined whether the MD suppressions in the ipsilateral and contralateral sides were at similar levels. As shown in Figures 7E, F, we computed the MDs difference between ipsilateral and contralateral arms for each neuron and showed that the number of neurons with a larger contralateral MD had a substantial increase (59 versus 79 among the 117 neurons for unimanual-4-direction versus bimanual movements; Figure 7F). Moreover, the mean of contralateral MDs became significantly larger than the ipsilateral ones in the bimanual task (Unimanual-4-direction: *mean* = 0.08, *median* = 0.05; Unimanual-8-direction: *mean* = 0.08, *median* = −0.05; Bimanual: *mean* = 1.66, *median* = 0.65; *p* < 0.001, *t*-test; right columns in Figures 7E, F). These results revealed that the bimanual coordination results in the suppression of bilateral modulation, especially for the ipsilateral side.

**FIGURE 8:**
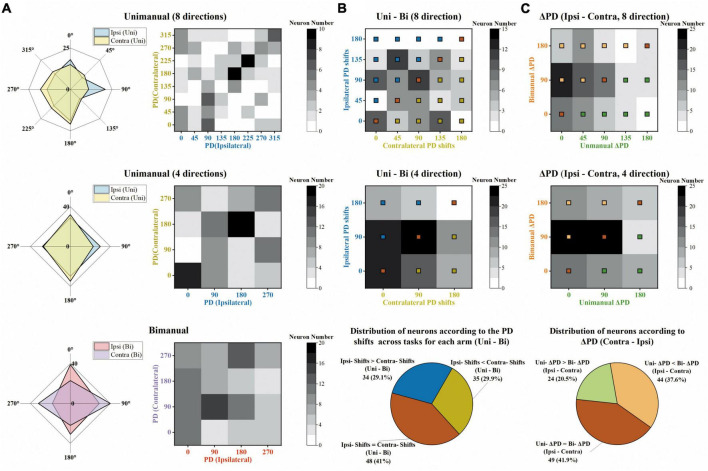
The same degree of preferred direction (PD) shifts for ipsilateral and contralateral sides across unimanual and bimanual tasks. **(A)** Top row Distribution of PDs for the ipsilateral (blue) and contralateral (yellow) arm movements, for the unimanual-8-direction data. Middle row and bottom row Same as in the top row, but for the unimanual-4-direction data (middle row) and the bimanual data (bottom row). **(B)** Top row and middle row Distribution of the PD shifts for ipsilateral (y-axis) and contralateral (x-axis) arms across unimanual versus bimanual tasks, for 8-direction (top row) and 4-direction data (middle row). Bottom row Classification of neurons according to the degree of ipsilateral and contralateral PD shifts across tasks, for 4-direction data in the middle row. While the bronze squares represent the neurons with the same ipsilateral and contralateral PD shifts across unimanual and bimanual tasks, blue squares and yellow squares represent those with larger ipsilateral PD shifts and larger contralateral PD shifts, respectively. **(C)** Top row and middle row Distribution of ΔPD during unimanual (x-axis) versus bimanual (y-axis) movements, for 8-direction (top row) and 4-direction data (middle row). Bottom row Classification of neurons according to the degree of ΔPD during the two tasks, for 4-direction data in the middle row. Similar to panel **(B)**, the neurons with the same ΔPD in the unimanual and bimanual tasks are marked with bronze squares, and other neurons with larger ΔPD in unimanual or in bimanual tasks are marked with green and orange squares, respectively. The above results show that the ipsilateral and contralateral sides exhibited the same degree of PD shifts across unimanual and bimanual tasks and these shifts contributed to the larger ΔPD in the bimanual versus unimanual tasks.

Next, we investigated how the PDs change across unimanual and bimanual tasks. As shown in Figure 8A, the ipsilateral and contralateral PDs showed great similarity in the unimanual task (top and middle rows) but lost alignment when switching to the bimanual task (bottom row). The heatmaps in Figure 8A depicted that 46.2% of the neurons exhibited the same PDs for bilateral arms in the unimanual task but decreased to 30% when using both arms simultaneously (diagonal panels; 54/117 and 34/117 neurons for unimanual-4-direction and bimanual data).

We further quantified how single units altered their PDs across unimanual and bimanual tasks. As shown in the heatmap in Figure 8B, the bimanual movements resulted in PD shifts on both sides, which altered by 67.7 ± 5.5° and 66.9 ± 6.07° for ipsilateral and contralateral arms (*mean* ± SEM). These indicated the same degree of neuronal PD shifts on both sides. The same results can also be obtained at the single-unit level as the classification results depicted in the pie chart in Figure 8B; 29.1% of neurons exhibited significant PD shifts on the ipsilateral side while 29.9% exhibited contralateral PD shifts.

We then wondered if these alternations in PDs for ipsilateral and contralateral arms result in changes in the bilateral relationship, which was quantified as the difference between contralateral and ipsilateral PDs (ΔPD) across unimanual versus bimanual tasks. For example, the neuron in Figure 3A had identical PDs when using either the ipsilateral or contralateral arm, (ΔPD = 0°) but its bilateral PDs were perpendicular in the bimanual task due to the PD shift in the contralateral side (ΔPD = 90°). In contrast, the second example neuron in Figure 3B exhibited opposite PDs in the unimanual task (ΔPD = 180°) and the difference decreased in the bimanual task (ΔPD = 90°) due to the changes in PDs of both arms. The distribution of the heatmaps in Figure 8C skewed mostly leftward, indicating the increased ΔPD across unimanual and bimanual tasks, which changed from 61.5 ± 6.0° to 83.8 ± 6.0° (*mean* ± SEM). For single units, the pie chart showed the statistical results that over half of neurons (58.1%) exhibited changes in ΔPD across unimanual and bimanual tasks; while 20.5% had larger ΔPD in the unimanual task, more neurons (37.6%) had larger changes in PD when switching to the bimanual task.

Generally, these results indicated that the bilateral directional tunings were highly correlated during unimanual movements, but were decorrelated when using both arms simultaneously. These changes can be attributed to the suppression of MDs for ipsilateral and contralateral arms, especially the ipsilateral side, and the increased bilateral PD shifts (ΔPD) across the two tasks contributed by similar-level PD shifts for both arms.

## 4. Discussion

In this study, we investigated how bimanual arms coordinate in human M1. Our results contribute to the findings (Donchin et al., 2002; Ames and Churchland, 2019) that the M1 outputs are not lateralized during unimanual movements, yet when using both arms simultaneously, the representation shifts to contralateral side. We found a reduction in the correlation between each arm’s representation across the unimanual and bimanual movements. Moreover, we identified that these changes can be attributed to the modification in tuning parameters, including MDs and PDs. These results provide further proof to the hypotheses of bimanual coordination obtained in physiological studies with non-human primates.

Our results are consistent with the suggestion that the ipsilateral modulations were separable (Donchin et al., 2002; Rokni et al., 2003; Diedrichsen et al., 2013; Ifft et al., 2013; Ames and Churchland, 2019; Berlot et al., 2019; Cross et al., 2020; Willett et al., 2020), suggesting a bilateral encoding of movement direction in the motor cortex. We demonstrated a BCI system with a 72 year-old patient with tetraplegia that was implanted with a microelectrode array in the arm area of his left M1 to record the neural signals during the motor imagery tasks. Most recorded neurons (117/136) in our study responded to both the unimanual and bimanual movements like the two typical neurons in Figure 3 which exhibited selectivity for the movement directions or the specific arm used. Actually, as depicted in Figure 4, neuronal responses exhibited separability for bilateral arms even across unimanual and bimanual tasks. These clinical results supported the mixed representation in the unilateral motor cortex and argued the opinion proposed by the classic electrical stimulation study (Leyton and Sherrington, 1917; Penfield and Boldrey, 1937; Sessle and Wiesendanger, 1982) that the limb movements were contralaterally mapped in the human body.

Our results support the idea that the neuronal responses in M1 were not lateralized during unimanual movements (Donchin et al., 2002; Ames and Churchland, 2019; Willett et al., 2020), and provided further evidence that neuronal responses biased to the contralateral side when switching to bimanual movements. While prior non-human studies with digit movements (Tanji et al., 1988; Aizawa et al., 1990) observed fewer ipsilateral-related activities in M1, it was disagreed by the fMRI studies with digit movements (Diedrichsen et al., 2013; Berlot et al., 2019) and other studies with arm movements (Donchin et al., 1998, 2002; Kermadi et al., 1998, 2000; Steinberg et al., 2002; Cisek et al., 2003; Rokni et al., 2003). In our study, like in non-human studies (Donchin et al., 2002; Ames and Churchland, 2019), we showed a similar proportion of neurons with either arm preference, indicating a similar level of neural responses for bilateral sides in the unimanual task. For the neural population, we proposed a similar level of prediction performance for the bilateral arms which also supported the non-lateralization. These results are consistent with the clinical study with movements across the whole-body parts regardless of using digits or arms (Willett et al., 2020). While the lateralization in the unimanual task has been investigated in several studies, we further proposed that the M1 outputs are biased to the contralateral side when using bimanual arms simultaneously at both the single-unit and population resolution. This change can be attributed to the one-third of neurons (33.3% of 117) like that in Figure 3B exhibiting an ipsilateral preference during unimanual movements but exhibiting inverted preference during bimanual movements.

Our results supported the findings (Steinberg et al., 2002; Rokni et al., 2003; Diedrichsen et al., 2013; Cross et al., 2020) that the ipsilateral and contralateral representations were correlated in M1 during unimanual movements, but few provided direct comparisons in the bimanual task. Our results of correlated directional tunings of bilateral arms were consistent with prior studies concerning the PDs of neuronal response properties, however, a non-human study with a cycling task concerning the temporal information of response properties proposed the limb-dependent hypothesis. A possibility is that the neurons might preserve direction selectivity when using either arm but delayed in conveying information from the ipsilateral side. In a near-infrared spectroscopy study (Shibuya et al., 2008), delayed oxygenation was observed in the ipsilateral M1, suggesting the time lagging in the ipsilateral tuning which might contribute to the decorrelated bilateral arm response found in the cycling task. For bimanual movements, while Rokni et al. (2003) proposed that the neurons lost their correlation in the bimanual task due to a substantial suppression in ipsilateral amplitudes and PD shifts, Cross et al. (2020) argued that there’s a small reduction in activation and little shifts in PDs for both arms during bimanual movements. Here, we provided detailed results about bilateral directional tunings and suggested that there is a significant decorrelation process between arms when switching to bimanual movements.

**FIGURE 9:**
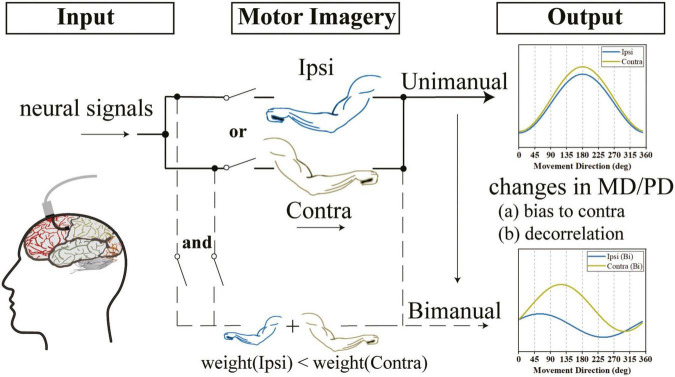
We observed that the outputs of the M1 area were not lateralized when using either arm and the directional tunings for ipsilateral and contralateral arms were highly correlated. However, when switching to bimanual movements, there was a decorrelation process and the outputs were biased to the contralateral side. We speculate that the changes in the bimanual relationship can be attributed to the alternation of tuning parameters, such as the increased ΔPD contributed by both the ipsilateral and contralateral sides and the significant suppression in bilateral MDs, especially the ipsilateral side.

Our conclusion provides further evidence that the changes in the bilateral relationship across unimanual and bimanual movements can be attributed to the suppression of MDs for both arms, especially the ipsilateral side (Figure 9). We supported the suggestion (Cross et al., 2020) that MDs of ipsilateral and contralateral sides were similar during unimanual movements, though other non-human studies for M1 (Steinberg et al., 2002; Rokni et al., 2003) and PMC (Willett et al., 2020) showed a nearly 50% reduction in ipsilateral modulations. For bimanual movements, our results supported the notion (Rokni et al., 2003) that the bimanual coordination contributed to a reduction in MDs for both arms, especially the ipsilateral side (Figure 7D); therefore, the contralateral MDs became significantly larger than the ipsilateral ones for neural ensembles (Figures 7E, F). However, these results argued the findings in a recent study with a postural perturbation task which proposed that the ipsilateral-related and contralateral-related modulation was suppressed at a comparable degree.

Our finding that the same-level PD shifts in bilateral sides contributed to the increased ΔPD is the other reason for the decorrelation process (Figure 9). Our results for similar changes in PDs for ipsilateral and contralateral arms across tasks (Figure 8B) argued the prior finding (Cisek et al., 2003; Rokni et al., 2003; Ifft et al., 2013; Cross et al., 2020) that the shifts in ipsilateral PDs were larger than contralateral ones. Our results also showed that the PD shifts in bilateral arms resulted in increased ΔPD (Figure 8C), which supported the results obtained in previous studies (Rokni et al., 2003). However, our results argued the findings in another study which found that most M1 neurons maintained their PDs when using both arms simultaneously (Cross et al., 2020).

A plausible explanation for these changes in the bilateral relationship across unimanual and bimanual tasks is that the corpus callosum induces a local inhibition in M1. With the callosal afferent inputs, single units’ response properties are altered to enable bimanual coordination which could be quantified as tuning parameters. In our study, the suppression in ipsilateral discharge frequency and bilateral PD shifts (ΔPD) are two factors of changes in the lateralization and decorrelation process. Indeed, previous clinical studies with callosal participants showed that the normal participants had a better performance in controlling bimanual arms simultaneously and revealed the essential role of the corpus callosum in bimanual coordination (Serrien et al., 2001; Kennerley et al., 2002).

Like other motor imagery studies (Aflalo et al., 2015; Alimardani et al., 2018), one limitation of our study would be that the recorded signals represent mixed visual cues and motor imagery, which we could not distinguish. Further study with no sensory cue or other modality like auditory cue could tell apart these effects. The visual cues, however, served as control variables and held constant throughout the unimanual and bimanual imagery tasks. Therefore, the changes observed across two tasks in single units and population decoding results cannot be simply explained by visual signals. It is also important to note that the subject (who cannot move his arms) in our study imagined all discrete arm movements, while most prior works were conducted with non-human primates (with freely moving arms) during attempted movement. The volitional state engaged in these paradigms are distinct (but hard to measure), which may contribute to the different findings among these works (Rastogi et al., 2020). Additionally, as the participant received no feedback (neither for the experimenters) during the experiment, the imaginary process depends on how he engaged in the task. However, to make sure the participant performed the task as close as we instructed, we used offline decoders to verify the intended movements are decodable and visualized the prediction results during the experiment. The decoding results in our paper were similar to those in prior works with attempted movements, revealing that our participant was at least engaged in the task.

Our study focused on the bimanual coordination in human left M1 of a participant with tetraplegia and proposed further evidence for how bilateral arms coordinate that obtained previously in non-human primates. These provided the possibility for bimanual control with only a single brain region under BCI systems and may contribute to the development of future clinical neuroprosthetics systems that enable paralyzed individuals to regain the bimanual upper arms function and perform natural behaviors of daily life. There’s a tendency for BCIs to control multiple limbs and perform precise interlimb tasks. As for future clinical applications, including more participants and adding blank control groups with simple observation or audio cues may help tease out the contamination of sensory input. Moreover, online decoding with closed-loop paradigms (Orset et al., 2021), intra-cortical microstimulation (ICMS) with tactile feedback (Collinger et al., 2018; Flesher et al., 2021), and effective training methods like adding biased feedback (Mladenovic et al., 2022) or training with error amplification (Marchal-Crespo et al., 2017) may help to improve the performance of BCI systems and thus facilitate the recovery of their arm function.

## Data availability statement

The original contributions presented in this study are included in the article/supplementary material, further inquiries can be directed to the corresponding authors.

## Ethics statement

The studies involving human participants were reviewed and approved by the Human Research Ethics Committee of the Second Affiliated Hospital of Zhejiang University School of Medicine (Protocol Number: 2019-15B). The patients/participants provided their written informed consent to participate in this study.

## Author contributions

DL and KX designed the study. DL, ZW, JL, LP, and FR contributed to the acquisition of data. DL, ZW, and FR performed the data analysis. DL, ZW, JL, YH, and KX interpreted the data. DL drafted the manuscript. JuZ, JiZ, YW, YH, and KX revised the manuscript. All authors contributed to the article and approved the submitted version.

## References

[B1] Aflalo T. Kellis S. Klaes C. Lee B. Shi Y. Pejsa K. (2015). Neurophysiology. Decoding motor imagery from the posterior parietal cortex of a tetraplegic human. *Science* 348 906–910. 10.1126/science.aaa5417 25999506 PMC4896830

[B2] Aizawa H. Mushiake H. Inase M. Tanji J. (1990). An output zone of the monkey primary motor cortex specialized for bilateral hand movement. *Exp. Brain Res.* 82 219–221. 10.1007/BF00230856 2257909

[B3] Alimardani M. Nishio S. Ishiguro H. (2018). “Brain-computer interface and motor imagery training: The role of visual feedback and embodiment,” in *Evolving BCI therapy - engaging brain state dynamics*, ed. Larrivee D. (London: IntechOpen). 10.1371/journal.pone.0161945

[B4] Ames K. C. Churchland M. M. (2019). Motor cortex signals for each arm are mixed across hemispheres and neurons yet partitioned within the population response. *Elife* 8:e46159. 10.7554/eLife.46159 31596230 PMC6785221

[B5] Berlot E. Prichard G. O’reilly J. Ejaz N. Diedrichsen J. (2019). Ipsilateral finger representations in the sensorimotor cortex are driven by active movement processes, not passive sensory input. *J. Neurophysiol.* 121 418–426. 10.1152/jn.00439.2018 30517048 PMC6397402

[B6] Bundy D. T. Szrama N. Pahwa M. Leuthardt E. C. (2018). Unilateral, 3D arm movement kinematics are encoded in ipsilateral human cortex. *J. Neurosci.* 38 10042–10056. 10.1523/JNEUROSCI.0015-18.2018 30301759 PMC6246886

[B7] Cisek P. Crammond D. J. Kalaska J. F. (2003). Neural activity in primary motor and dorsal premotor cortex in reaching tasks with the contralateral versus ipsilateral arm. *J. Neurophysiol.* 89 922–942. 10.1152/jn.00607.2002 12574469

[B8] Collinger J. L. Gaunt R. A. Schwartz A. B. (2018). Progress towards restoring upper limb movement and sensation through intracortical brain-computer interfaces. *Curr. Opin. Biomed. Eng.* 8 84–92.

[B9] Collinger J. L. Wodlinger B. Downey J. E. Wang W. Tyler-Kabara E. C. Weber D. J. (2013). High-performance neuroprosthetic control by an individual with tetraplegia. *Lancet* 381 557–564. 23253623 10.1016/S0140-6736(12)61816-9 PMC3641862

[B10] Cross K. P. Heming E. A. Cook D. J. Scott S. H. (2020). Maintained representations of the ipsilateral and contralateral limbs during bimanual control in primary motor cortex. *J. Neurosci.* 40 6732–6747. 10.1523/JNEUROSCI.0730-20.2020 32703902 PMC7455209

[B11] Diedrichsen J. Wiestler T. Krakauer J. W. (2013). Two distinct ipsilateral cortical representations for individuated finger movements. *Cereb. Cortex* 23 1362–1377. 10.1093/cercor/bhs120 22610393 PMC3643717

[B12] Donchin O. Gribova A. Steinberg O. Bergman H. Vaadia E. (1998). Primary motor cortex is involved in bimanual coordination. *Nature* 395 274–278. 9751054 10.1038/26220

[B13] Donchin O. Gribova A. Steinberg O. Mitz A. R. Bergman H. Vaadia E. (2002). Single-unit activity related to bimanual arm movements in the primary and supplementary motor cortices. *J. Neurophysiol.* 88 3498–3517. 12466464 10.1152/jn.00335.2001

[B14] Flesher S. N. Downey J. E. Weiss J. M. Hughes C. L. Herrera A. J. Tyler-Kabara E. C. (2021). A brain-computer interface that evokes tactile sensations improves robotic arm control. *Science* 372 831–836. 34016775 10.1126/science.abd0380 PMC8715714

[B15] Gardner E. P. Putrino D. F. Chen Van Daele J. (2022). Neural representation in M1 and S1 cortex of bilateral hand actions during prehension. *J. Neurophysiol.* 127 1007–1025. 10.1152/jn.00374.2021 35294304 PMC8993539

[B16] Georgopoulos A. P. Kalaska J. F. Caminiti R. Massey J. T. (1982). On the relations between the direction of two-dimensional arm movements and cell discharge in primate motor cortex. *J. Neurosci.* 2 1527–1537. 7143039 10.1523/JNEUROSCI.02-11-01527.1982 PMC6564361

[B17] Handelman D. A. Osborn L. E. Thomas T. M. Badger A. R. Thompson M. Nickl R. W. (2022). Shared control of bimanual robotic limbs with a brain-machine interface for self-feeding. *Front. Neurorobot.* 16:918001. 10.3389/fnbot.2022.918001 35837250 PMC9274256

[B18] Hochberg L. R. Bacher D. Jarosiewicz B. Masse N. Y. Simeral J. D. Vogel J. (2012). Reach and grasp by people with tetraplegia using a neurally controlled robotic arm. *Nature* 485 372–375. 22596161 10.1038/nature11076 PMC3640850

[B19] Hochberg L. R. Serruya M. D. Friehs G. M. Mukand J. A. Saleh M. Caplan A. H. (2006). Neuronal ensemble control of prosthetic devices by a human with tetraplegia. *Nature* 442 164–171. 16838014 10.1038/nature04970

[B20] Ifft P. J. Shokur S. Li Z. Lebedev M. A. Nicolelis M. A. (2013). A brain-machine interface enables bimanual arm movements in monkeys. *Sci. Transl. Med.* 5:210ra154. 10.1126/scitranslmed.3006159 PMC3967722 24197735

[B21] Kennerley S. W. Diedrichsen J. Hazeltine E. Semjen A. Ivry R. B. (2002). Callosotomy patients exhibit temporal uncoupling during continuous bimanual movements. *Nat. Neurosci.* 5 376–381. 10.1038/nn822 11914724

[B22] Kermadi I. Liu Y. Rouiller E. M. (2000). Do bimanual motor actions involve the dorsal premotor (PMd), cingulate (CMA) and posterior parietal (PPC) cortices? Comparison with primary and supplementary motor cortical areas. *Somatosens. Mot. Res.* 17 255–271. 10.1080/08990220050117619 10994596

[B23] Kermadi I. Liu Y. Tempini A. Calciati E. Rouiller E. M. (1998). Neuronal activity in the primate supplementary motor area and the primary motor cortex in relation to spatio-temporal bimanual coordination. *Somatosens. Mot. Res.* 15 287–308. 10.1080/08990229870709 9875547

[B24] Lebedev M. A. Nicolelis M. A. (2017). Brain-machine interfaces: From basic science to neuroprostheses and neurorehabilitation. *Physiol. Rev.* 97 767–837. 10.1152/physrev.00027.2016 28275048

[B25] Leyton A. S. F. Sherrington C. S. (1917). Observations on the excitable cortex of the chimpanzee, orang-utan, and gorilla. *Q. J. Exp. Physiol.* 11 135–222.

[B26] Ma C. Ma X. Fan J. He J. (2017). Neurons in primary motor cortex encode hand orientation in a reach-to-grasp task. *Neurosci. Bull.* 33 383–395. 10.1007/s12264-017-0126-1 28389871 PMC5567557

[B27] Marchal-Crespo L. Michels L. Jaeger L. Lopez-Oloriz J. Riener R. (2017). Effect of error augmentation on brain activation and motor learning of a complex locomotor task. *Front. Neurosci.* 11:526. 10.3389/fnins.2017.00526 29021739 PMC5623679

[B28] Mladenovic J. Frey J. Pramij S. Mattout J. Lotte F. (2022). Towards identifying optimal biased feedback for various user states and traits in motor imagery BCI. *IEEE Trans. Biomed. Eng.* 69 1101–1110. 10.1109/TBME.2021.3113854 34543189

[B29] Mooshagian E. Snyder L. H. (2018). Spatial eye-hand coordination during bimanual reaching is not systematically coded in either LIP or PRR. *Proc. Natl. Acad. Sci. U.S.A.* 115 E3817–E3826. 29610356 10.1073/pnas.1718267115 PMC5910835

[B30] Mooshagian E. Wang C. Holmes C. D. Snyder L. H. (2018). Single units in the posterior parietal cortex encode patterns of bimanual coordination. *Cereb. Cortex* 28 1549–1567. 10.1093/cercor/bhx052 28369392 PMC5907348

[B31] Orset B. Lee K. Chavarriaga R. Millan J. D. R. (2021). User adaptation to closed-loop decoding of motor imagery termination. *IEEE Trans. Biomed. Eng.* 68 3–10. 10.1109/TBME.2020.3001981 32746025

[B32] Pandarinath C. Nuyujukian P. Blabe C. H. Sorice B. L. Saab J. Willett F. R. (2017). High performance communication by people with paralysis using an intracortical brain-computer interface. *Elife* 6:e18554. 10.7554/eLife.18554 PMC5319839 28220753

[B33] Penfield W. Boldrey E. (1937). Somatic motor and sensory representation in the cerebral cortex of man as studied by electrical stimulation. *Brain* 60 389–443.

[B34] Rastogi A. Vargas-Irwin C. E. Willett F. R. Abreu J. Crowder D. C. Murphy B. A. (2020). Neural representation of observed, imagined, and attempted grasping force in motor cortex of individuals with chronic tetraplegia. *Sci. Rep.* 10:1429. 10.1038/s41598-020-58097-1 31996696 PMC6989675

[B35] Rokni U. Steinberg O. Vaadia E. Sompolinsky H. (2003). Cortical representation of bimanual movements. *J. Neurosci.* 23 11577–11586. 14684860 10.1523/JNEUROSCI.23-37-11577.2003 PMC6740952

[B36] Serrien D. J. Nirkko A. C. Wiesendanger M. (2001). Role of the corpus callosum in bimanual coordination: A comparison of patients with congenital and acquired callosal damage. *Eur. J. Neurosci.* 14 1897–1905. 10.1046/j.0953-816x.2001.01798.x 11860484

[B37] Sessle B. J. Wiesendanger M. (1982). Structural and functional definition of the motor cortex in the monkey (*Macaca fascicularis*). *J. Physiol.* 323 245–265. 10.1113/jphysiol.1982.sp014071 7097574 PMC1250355

[B38] Shibuya K. Sadamoto T. Sato K. Moriyama M. Iwadate M. (2008). Quantification of delayed oxygenation in ipsilateral primary motor cortex compared with contralateral side during a unimanual dominant-hand motor task using near-infrared spectroscopy. *Brain Res.* 1210 142–147. 10.1016/j.brainres.2008.03.009 18423579

[B39] Simeral J. D. Kim S. P. Black M. J. Donoghue J. P. Hochberg L. R. (2011). Neural control of cursor trajectory and click by a human with tetraplegia 1000 days after implant of an intracortical microelectrode array. *J. Neural Eng.* 8:025027. 10.1088/1741-2560/8/2/025027 21436513 PMC3715131

[B40] Steinberg O. Donchin O. Gribova A. Cardosa De Oliveira S. Bergman H. Vaadia E. (2002). Neuronal populations in primary motor cortex encode bimanual arm movements. *Eur. J. Neurosci.* 15 1371–1380. 11994131 10.1046/j.1460-9568.2002.01968.x

[B41] Tanji J. Okano K. Sato K. C. (1988). Neuronal activity in cortical motor areas related to ipsilateral, contralateral, and bilateral digit movements of the monkey. *J. Neurophysiol.* 60 325–343. 10.1152/jn.1988.60.1.325 3404223

[B42] Truccolo W. Friehs G. M. Donoghue J. P. Hochberg L. R. (2008). Primary motor cortex tuning to intended movement kinematics in humans with tetraplegia. *J. Neurosci.* 28 1163–1178. 18234894 10.1523/JNEUROSCI.4415-07.2008 PMC6671402

[B43] Willett F. R. Deo D. R. Avansino D. T. Rezaii P. Hochberg L. R. Henderson J. M. (2020). Hand knob area of premotor cortex represents the whole body in a compositional way. *Cell* 181 396–409.e26. 10.1016/j.cell.2020.02.043 32220308 PMC7166199

[B44] Wodlinger B. Downey J. E. Tyler-Kabara E. C. Schwartz A. B. Boninger M. L. Collinger J. L. (2015). Ten-dimensional anthropomorphic arm control in a human brain-machine interface: Difficulties, solutions, and limitations. *J. Neural Eng.* 12:016011. 10.1088/1741-2560/12/1/016011 25514320

